# The perceived value of local knowledge tourism: dimension identification and scale development

**DOI:** 10.3389/fpsyg.2023.1170651

**Published:** 2023-08-10

**Authors:** Hailin Zhang, Jinbo Jiang, Jinsheng (Jason) Zhu

**Affiliations:** ^1^Department of Tourism Management, South China University of Technology, Guangzhou, China; ^2^ASEAN Tourism Research Base, Guilin Tourism University, Guilin, China

**Keywords:** local knowledge tourism, perceived value, value dimension, scale development, tourist experiences

## Abstract

**Introduction:**

Local knowledge tourism encompasses the rich cultural heritage, historical narratives, and traditional practices of a specific destination. Despite its significance in enhancing the tourist experience, there is a dearth of research examining the subjective perceptions and values of visitors engaging in local knowledge tourism. Consequently, there is a pressing need to explore the composition of perceived tourist values in this unique context.

**Methods:**

Due to the exploratory nature of this research, a constructivist grounded theory and content analysis are applied to analyze the data.

**Results:**

This study identifies and conceptualizes five distinct dimensions of perceived values in local knowledge tourism: functional value, emotional value, social value, cognitive value, and self-actualization value. Furthermore, an 18-item scale is developed to measure these dimensions quantitatively.

**Discussion:**

This research makes several significant contributions: (1) it expands the scope of perceived value research within the tourism domain and enhances our understanding of the tourist experience in local knowledge tourism; (2) it provides a reliable instrument for future quantitative investigations into the behavior and mindset of local knowledge tourists; and (3) it offers theoretical foundations and practical insights for destination managers seeking to develop tourism products tailored to the preferences and expectations of local knowledge tourists.

## Introduction

Local knowledge refers to the traditional knowledge, values, skills, beliefs, and philosophy that communities have developed through long-term interactions with their natural and cultural environments (Lenzerini, 2011). This diverse and valuable knowledge has profound social, cultural, ecological, scientific, and economic significance, reflecting the rich diversity of human civilization (Hill et al., 2020). Local knowledge is increasingly recognized as a significant tourist attraction and plays a crucial role in product development within the tourism industry (Gato et al., 2022). To address sustainability and social justice in the tourist industry, it is essential to recognize the diversity of cultures and the inherent multifaceted modalities of knowledge. However, there is a need to further explore and understand the structure, characteristics, and assessment of perceived value in the context of local knowledge tourism to promote sustainability and social justice in the tourism sector (Ramos, 2015). Local knowledge tourism is developed focusing on the elements of knowledge in heritage tourism and to facilitate dialogue and exchange between diverse cultures (Moayerian et al., 2022). As such, it is a constructive response to the need of strengthening tourist experience in the era of cultural and tourism integration (Yuan et al., 2022). Unlike heritage tourism which merely promotes superficial historical landscape viewing and experience, local knowledge tourism stresses in-depth visitor participation and knowledge development. Existing research has not given sufficient attention to visitors’ subjective impression and experience of an attraction’s worth in local knowledge tourism, and the structure, features, and assessment of perceived value must be elucidated.

The definition of perceived tourist value is derived from the marketing concept of customer values and was reintroduced into tourism studies in the 1990s (Overstreet, 1993). Since the dawn of the 21st century, the concept has been extended to tourist consumer behavior and destination marketing research. It has also emerged as a new prominent research topic, following in the footsteps of quality management and visitor satisfaction (Li et al., 2009; Zhu et al., 2021). This notion offers a perfect vantage point from which to comprehend the tourists’ all-encompassing assessments of their travel experiences within the framework of the consumption-based model of local knowledge tourism. The investigation of tourist perceived values will contribute to a deeper comprehension of tourist behavioral characteristics, consumption behavior, and consumption psychology, thereby furnishing a theoretical foundation for the development of products and a marketing strategy for local knowledge tourism.

And thus, the current research aims to address this gap and highlight the significance of local knowledge in enhancing the tourist experience. The current research intended to address the questions as follows, (1) What are the key components and dimensions of perceived value in local knowledge tourism? (2) How does the perceived value of local knowledge tourism contribute to sustainable tourism development and social justice? (3) What fundamental dimensions and scales can be implemented to enhance and promote the perceived value of local knowledge tourism? This exploratory research, conducted in the context of Guilin, China, employs a grounded theory approach to identify the multidimensional structure of perceived value in local knowledge tourism and develop a rigorous scale. By applying a stringent scientific procedure, this study aims to provide theoretical support and practical guidance for the development of local knowledge tourism, addressing a gap in the existing literature.

The paper is structured as follows: the introduction provides an overview of the significance of local knowledge tourism and the importance of perceived value. The literature review delves into the concepts of local knowledge, perceived value, and their relevance to the tourism industry. The methodology section describes the case study approach and the grounded theory utilized to identify the structure of perceived value in local knowledge tourism. The findings and analysis section present the results of the study, including the multidimensional structure and components of perceived value. The discussion section interprets the findings, highlighting their theoretical and practical implications. Finally, the conclusion summarizes the key insights and suggests future research directions in local knowledge tourism and perceived value.

## Literature review

### The theory of local knowledge

Geertz (1974, p. 19), an American anthropologist, is attributed with introducing the idea of “local knowledge” as a central notion in interpretative anthropology; nevertheless, he did not provide a precise term for the concept. Local knowledge has been characterized in a variety of ways, depending on the setting and the goals of various researchers and organizations. For instance, the United Nations’ Local and Indigenous Knowledge Systems Program (LINKS), which aims to support the preservation and transmission of collective memory and cultural heritage, defines that understandings, capacities, and philosophies formed by communities with extensive histories of engagement with their natural environs are referred to as local knowledge (UNESCO, 2017). Local knowledge plays an essential role in a cultural network that also includes linguistic systems, categorization schemes, techniques for making use of resources, social interactions, and rituals and spiritual activities. Local knowledge, on the other hand, is a form of peripheral and unofficial knowledge held by indigenous people. This stands in contrast to the universal scientific knowledge centered on the West. Broadly speaking, it is the sum of material and cultural achievements accumulated by people in a certain region during their historical development, involving every aspect of life that may include local economic development, science and technology, social values (SVs), religious beliefs, culture and arts, social customs, lifestyles, and social codes of conduct (Correia et al., 2013; Lepore et al., 2021). It is not always related to specific daily life, but also contains abstract thoughts, philosophies, and insights, which are comprehensive knowledge and technology about ecology, geography, or society that revitalize the locale (Gao and Wu, 2017; Zhu and Siriphon, 2019).

### The theory of local knowledge tourism

Local knowledge can highlight the diversity and equality of different cultures (see Table 1 below). The mysterious local knowledge is inherently attractive to the general public, by which such knowledge is related to the cultural economy (Zhang and Liu, 2012), festivals creation (Chaiboonsr et al., 2022), coproduction of histories (Glover, 2008), as well as ethnic tourism development issues (Chatzopoulou et al., 2019). Integrating local knowledge into the design of participatory tourism can highly promote the quality of tourism development, participation of local community in tourism planning and operation, as well as heritage preservation (Pongponrat, 2011; Pongponrat and Chantradoan, 2012; de Bruin and Jelinčić, 2016). Deep experience of local knowledge in a destination, for instance, local engagements (Sofield et al., 2017; Rahmanita, 2018) and indigenous tourism experiences (Butler, 2017) will contribute to the tourist experience and lead to in-depth tourism. A consensus has been reached that enhancing cultural integration and tourist experience in destination management has become a significant trend for tourism planning and products development (Beritelli et al., 2007; Halkier, 2014; Pechlaner et al., 2015). Based on the above discussion, this study defined local knowledge tourism as a type of heritage tourism that utilize local knowledge of a destination as its essence of tourism attraction, aiming to conserve the traditional cultural heritage and provide an in-depth and participatory cultural tourism experience and knowledge acquisition opportunity to tourists. From the perspective of the tourist, one of the main goals or motivations is to gain local indigenous knowledge of the destination. Since local knowledge encompasses the knowledge and practices contextualized in the local daily life experiences, such tourist activities can offer a deeper cultural experience compared to other types of heritage tourism.

**TABLE 1:** Scale examples of tourist perceived value.

References	Scenarios	Measurement of perceived value
Sheth et al., 1991	Commodities	5 dimensions: functional value, social value, emotional value, conditional value, and epistemic value
Pi et al., 2016	Tourist destination	4 dimensions: functional value, social value, emotional value, and brand value
Ma and Bao, 2012	Traditional event tourism	7 dimensions: cultural epistemic value, hedonic value, social value, price and convenience value, service value, conditional value and overall value, environmental value, and functional value
Petrick, 2004	Cruise tourism	5 dimensions: quality value, emotional value, cost value, behavior cost value, and reputation value
Kim and Thapa, 2018	Eco-tourism	4 dimensions: quality value, emotional value, cost value, and social value
Rasoolimanesh et al., 2016	Community-based homestay tourism	3 dimensions of inclusive of functional, emotional, and social value
Na et al., 2019	Tourist destination	3 core dimensions: functional value, hedonic value, and symbolic value, and 8 subcategories: relaxation, healing, communication, and social prestige

Data source: this study.

### The theory of tourist perceived value

Customer perceived value was first discussed in the field of marketing and refers to how customers feel about a product or service with regard to perceived quality, internal and external features of the product, and other psychological benefits (Zeithaml, 1988). It has been defined as a customer’s assessment of the trade-offs between the benefits and sacrifices realized in selecting a given product from the options available in the market (Sánchez-Fernández and Iniesta-Bonillo, 2007; Lim, 2013; Behnam et al., 2023). Perceived costs consist of monetary costs and non-monetary costs such as costs of time, physical efforts or life style changes that one has to pay to acquire the product or service (Snoj et al., 2004). As a special form of customer perceived value, tourist perceived value is the emotional and psychological benefits generated by a series of interaction between tourists and the outside world. It is an important research dimension to grasp tourists’ perception and experience of tourist destinations (Bao and Xie, 2019). It is defined as the trade-off between perceived gains and losses of tourists in a specific tourism context and it is an overall evaluation of tourist products or services (Luo et al., 2020; Zhu et al., 2023). Perceived value is deemed to have great impact on tourist preference, satisfaction, and loyalty, for example, high level of perceived value will lead to the repurchase of the products and service (Chen and Chen, 2010). Therefore, measuring the perceived value of tourists may contribute to a better understanding of tourists’ consumption behavior and psychology, as well as provide theoretical basis for tourism marketing (Biao et al., 2020). The circumstances, quality, and attributes of tourism products play a significant role in determining the tourist perceived value. Meanwhile, it is also influenced by subjective elements such as preference, attitude, physiological condition, demand, and motivation (Woodruff, 1997; Zhang et al., 2018). Compared to consumption activities in other commercial sectors, tourist consumption is more complicated and diverse, and the perception of tourist worth has plural and structural dimensions. The perceived value of a tourist destination contains functional value (FV), hedonic value, and symbolic worth (Xie and Li, 2009). Due to the intangible, diachronic, and interactive features of tourism products and services, tourists tend to perceive the value of tourism experiences from a holistic perspective considering multiple elements such as a product, service, and destination (Huang and Huang, 2007).

### Dimensions and measurement of tourist perceived value

Customers perceived value are mainly assessed in the unidimensional approach (Sánchez-Fernández and Iniesta-Bonillo, 2007) and the multidimensional approach (Loureiro et al., 2012). The unidimensional approach emphasizes economic utility apart from the trade-offs between the benefits such as customer utility and sacrifices such as price, time, effort, etc. (Bunghez, 2016; Yin et al., 2017). A typical practice is to incorporate the tourist perceived value as an independent variable into the model of tourist satisfaction or loyalty (Chenini and Touaiti, 2018). To overcome the limitations of the unidimensional approach that ignores various aspects of a person’s emotional state and external conditions, the multidimensional approach was proposed to explore the factors underlying the phenomenon (Sheth et al., 1991; Sweeney and Soutar, 2001). Using the multidimensional approach, Sheth et al. (1991) identified five dimensions of customer perceived value that include social, emotional, functional epistemic, and conditional value. Sweeney and Soutar (2001) synthesize previous studies (e.g., Groth, 1995; Grönroos, 1997; de Ruyter et al., 1998) to develop the four-dimension PERVAL scale in which the emotional value (EV), for example, represents the utility derived from the feelings or affective states that a product generates; and SV refers to the utility derived from the product’s ability to enhance social relationship.

To assess the tourist perceived values, scales have been modified and developed in different tourism contexts, such as the five-dimensional SERV-PERVAL scale that encompasses quality, monetary price, non-monetary price, reputation, and emotional response for cruise tourism (Petrick, 2004). Andrades and Dimanche (2018) analyze tourist value perception from the perspective of psychology and developed a scale of co-creating tourists’ experiential value at tourist attractions. Furthermore, related scales may involve the overall perception of a tourist destination, ecotourism, traditional event tourism, and cruise tourism. These studies might expand our understanding of consumer value in tourism and serve as a reference for the current inquiry, which will provide a reliable tool for quantitative study in the context of local knowledge tourism.

## Grounded theory analysis and value dimensions identification

### Research methods

Due to the exploratory nature of this research, constructivist grounded theory and content analysis are applied to analyze the data collected. Grounded theory is useful to develop theory based on the analysis of systematically collected data and through interaction with study participants (Mills et al., 2006). To realize the ultimate goal of theoretical construction with high reliability and validity, further to the primary data from the semi-structured interviews, secondary data are extracted from sources like online travelogue and news report to form a triangle cross validation. In the text encoding and follow-up study, review and advice are also sought from a group of experts for item purification. In the verification part, SPSS26.0 and AMOS24.0 were used for confirmatory factor analysis, reliability and validity analysis, and competitive factor analysis.

Guilin city, China, is chosen as the study case because it is a world-famous tourist destination and a recognized national historical and cultural city, as designated by the Chinese central government. In addition, Guilin is home to a dozen ethnic minorities, including Zhuang, Dong, Miao, and Yao. With extensive natural, cultural, and tourist resources, Guilin tourism contains rich local knowledge in a variety of forms, from traditional agricultural lifestyle to genuine ethnic culture (Zhu and Siriphon, 2019), making it an appropriate case study for this investigation.

### Data collection

The first step is to solicit the views of the respondents regarding four aspects of their local knowledge tourism experience: tourism motivation, experience process, experience value perception, personal feelings, and gain. From March to May 2022, semi-structure interviews were conducted in five local knowledge tourism experience zones in Guilin: the East West Alley, Longsheng Terrace Field, Guangxi Ethnic Tourism Museum, West Street of Yangshuo, and venue of the Liusanjie Impression Show. Each interview lasted about 20–30 min, and the whole process was voice recorded with the permission of the interview participants. According to the selection criteria, the participants needed to have conducted a local knowledge tourism in Guilin as defined by this study. A total of 22 participants were recruited through snowball sampling and their demographic profile is shown in Table 2.

**TABLE 2:** Profile of the respondents (*N* = 22).

No.	Gender	Age	Occupation	Origin	No.	Gender	Age	Occupation	Origin
T1	F	21–30	Student	Guangxi	T2	M	21–30	Student	Jiangsu
T3	M	41–50	Company employee	Beijing	T4	F	21–30	Company employee	Guangxi
T5	F	31–40	Teacher	Guizhou	T6	F	21–30	Teacher	Guangxi
T7	M	31–40	Teacher	Guizhou	T8	F	31–40	Researcher	Guangxi
T9	F	41–50	Self-employed	Guangxi	T10	M	31–40	Company employee	Guangxi
T11	F	51–60	Company employee	Guangxi	T12	M	31–40	Company employee	Guangxi
T13	F	21–30	Self-employed	Guangxi	T14	M	51–60	Retired	Guangxi
T15	M	21–30	Student	Hunan	T16	F	21–30	Student	Guangxi
T17	M	21–30	Self-employed	Guangxi	T18	F	21–30	Student	Guangxi
T19	F	41–50	Company employee	Guangxi	T20	M	21–30	Company employee	Guangxi
T21	F	31–40	Self-employed	Shanghai	T22	F	31–40	Doctor	Guangxi

Data source: this study.

### Coding and identifying the value dimensions

Using a method known as thematic analysis, the data were then evaluated and examined. Using the professional qualitative research software NVivo 11.0 for data coding, the thematic analysis has identified 19 concepts and 5 dimension of the tourism perceived values of local knowledge, including the FV, EV, SV, cognitive value (CV), and self-actualization value (SAV) as shown in Tables 3, 4.

**TABLE 3:** Example of open coding of local knowledge tourism perceived value.

Concepts	Source	Original text sample
A1 Daily application	Interview participants; travelogue	1. Local people know how to adapt to local conditions. This kind of traditional stilted house^1^ design can save land and be built along the slope up. It is warm in winter and cool in summer, and the ground floor is used to keep livestock down here. (T10) 2. Look at the hair of the aunt! She is 68 years old, but she has not one white hair on her head. In Yao Long Hair Village, women cut their hair only three times in their whole life, at birth, marriage, and death. The aunt said that their habit is to wash their hair with a kind of special cold water that has been used to wash rice all year, so their hair is always so black and shiny. (The MFW 2)
A2 Physical and mental pleasure	Interview participants; travelogue	1. The Zhuang ethnic people in Longji is a representative of the Northern Zhuang, with unique costumes and customs. Here, we can see the ancient Zhuang folk dances and costumes that has been very well protected. We can also enjoy the beautiful Zhuang folk songs and experience their life scenes such as how they repair their wooden houses. It is so pleasant and refreshing. (T6) 2. We went to various scenic spots on the second day. Everyone was so happy as we could feel and experience the ethnic and local characteristics. (T8)
A3 Perceive the cost	Interview participants	1. At first, we took a bus, because the local terrain was special and difficult. We had to get off and walk in the middle of the journey, our bus could not get to the village. We need to change to a small local car to get to the final destination at the end. (T6) 2. The Longji terraced fields are beautiful but tiring to climb. (T2) 3. It is not easy to get around here, quite challenging and hard. (T3)
A4 Feel the atmosphere	Interview participants; travelogue; news report	1. Children in the kindergarten are now required to wear ethnic costumes during ethnic costumes during the Zhuang 3 March Festival, giving us a good sense of ritual and a feeling of the festival. (T7) 2. Throwing the embroidered ball is a traditional sport popular with the Zhuang people, and it is also conducted as a ritual to seek a husband. The plot of show can really motivate audience’s emotions. (MFW 8) 3. In the live broadcast at the East and West Alley, the hostess showed the special local cuisine, traditional culture, and knowledge of Guilin to the audience online, all participants, regardless of their origin and location, can feel the strong festive atmosphere. (Sohu 2)
A18 Realize your personal aspirations	Interview participants	1. I have always been interested in ethnic culture and knowledge. Today I came here to experience these ethnic cultures with my own eyes, which is more vivid and direct than from books. I guess I got what I wanted. (T16) 2. I have seen something very special here; I feel like my life has become a little richer. (T12)
A19 Individual potential	Interview participants; travelogue	1. Only when we reached the top of the mountain can we truly feel the ritual sense of measuring the land with our own footsteps and the sense of conquest when we look back. (T12) 2. (The local culture study tour in the ancient Dongli Village of Guilin) It was the first time for Xiao Xiao to travel without her family, I always believe that the potential of children is unlimited, we should believe and support them, it is really an unforgettable journey of research and learning. (MFW 6)

Data source: this study.

^1^The stilt house is a traditional residence house for ethnic minority such as Zhuang, Yao, and Miao in southwest of China.

**TABLE 4:** Results of axial coding and selective coding.

Main category	Corresponding subcategory	Value connotation
Functional value (FV)	Daily usage	Perceive the basic utility, functionality and practicability of local knowledge in daily life and the cost to attain such tourism experience
	Physical and mental pleasure	
	Perceive the cost	
Emotional value (EV)	Feel the atmosphere	Obtain the emotions such as happiness, pleasure, attachment, love, admiration, shock, nostalgia, comfort concerned with local knowledge in the tourist experience
	Inspire association	
	Perception and thrill	
	Identity and attachment	
Social value (SV)	Make friends	Local knowledge tourism experience helps one make more friends, deepen the host–tourist interaction and attain other social purpose
	Host–guest interaction	
	Deepen the emotion	
	Obtain the information	
Cognition value (CV)	Experience the culture	Acquire knowledge about the unique ecology, nature, humanity, history, art, religion, and other aspects of the tourist destination as a result of the local knowledge tourism experience
	Understand the nature	
	Understand the society	
	Self-cognition	
Self-accomplishment value (SAV)	Self-promotion	Realize your personal dream, aspirations, wishes, and interests, and increase your social prestige, knowledge, and spirit because of the local knowledge tourism experience
	Social Prestige	
	Realize your personal aspirations	
	Individual potential	

Data source: this study.

## Scale development

### Item design

The initial scale items of this study were developed from multiple sources such as researchers, experts, and literature. Compared to the mere use of the inductive and deductive methods, the mixed method with multiple item sources will have a higher level of validity (Zhu et al., 2022; Lyu et al., 2023). Based on the 19 subcategories defined by the grounded theory analysis and some well-accepted tourism perceived value scales, a total of 25 initial scale items were developed. Following Delphi method, experts from Guilin Culture and Tourism Bureau, Guangxi Ethnic Tourism Museum, and Guilin Tourism University were invited to review and purify the initials items on the principle of conciseness, completeness, and correctness. As a result, 6 items were deleted, and remaining items refined in wording. The number of items is consistent with those of the formal questionnaire.

### The pilot survey to develop the scale

#### Investigation process and sample analysis

The pilot survey questionnaire is divided into three parts. The first part of the questionnaire includes a brief description of the questionnaire and the concept of local knowledge tourism with examples and photos. The second part is the five-dimension measurement adopting the 5-level Likert scale with “1” meaning “strongly disagree” and “5” meaning “strongly agree.” The third part addresses the personal information of the correspondent. According to Klein et al. (1994), the sample size should be 10–20 times the number of measured items. The pilot study was carried out online between April and May 2022 to reduce the hazards associated with person-to-person interaction in COVID-19. A key recruitment requirement for respondents is that they must have a local knowledge tourism experience as defined by this study in Guilin within the last 12 months. To ensure the reliability and validity, questionnaires that took less than 120 s to complete and all choice are the same are deemed to be invalid. As such, 183, or 87.6% of the valid questionnaires were finally collected out of the total 209 distributed.

As shown in Table 5, 56.8% of the respondents are female. A total of 82.5% are 21–60 years old, while those below 20 and above 61 accounted for 5.5 and 2.2%, respectively. As for occupations, civil servants represent 22.4%, followed by staff members in cultural, education, science and technology sectors (18.0%), enterprise employees (13.1%), and students (17.5%). Regarding education background, 50.8% are college/undergraduate students. To sum up, the demographic characteristics of the pilot survey respondents are basically in line with the real situation of local knowledge tourists.

**TABLE 5:** Profile of the pilot survey respondents (*N* = 183).

Variable	Category	Frequency	Percentage
Gender	M	79	43.2
	F	104	56.8
Age	Below 20	10	5.5
	21–30	53	29.0
	31–40	43	23.5
	41–50	51	27.9
	51–60	22	12.0
	Above 61	4	2.2
Occupation	Civil servants	41	22.4
	Company staff	24	13.1
	Student	32	17.5
	Teacher/researcher/ medical worker	33	18.0
	Others	53	29.0
Education	High school and college age students	1	0.5
	High school seniors	8	4.4
	Higher diploma/ bachelor	93	50.8
	Master and above	80	43.7
	Others	1	0.5
Monthly income (yuan) Note: 1 USD = 7.1 yuan	Below 2,000	30	16.4
	2,000–4,999	36	19.7
	5,000-7,999	50	27.3
	8,000-9,999	22	12.0
	Above 10,000	45	24.6

Data source: this study.

#### Descriptive statistical analysis

As illustrated in Table 6, the mean values of FV, EV, SV, CV, and SAV were 4.30, 4.04, 4.15, 4.19, and 3.16, respectively. Although the score of SAV was relatively low as compared to other value dimensions, which can be explained by the classic theory of Maslow’s needs hierarchy. Self-actualization lies at the top of the hierarchy of needs; hence, it is the most complex and difficult to attain. Secondly, the kurtosis and skewness of data are used for testing whether samples are normally distributed, which indicates the universality of samples. The data skewness coefficient of each measurement element ranges between −1.903 and 0.156 with the absolute value smaller than 2, and the kurtosis coefficient is between −0.643 and 5.443 with the absolute value less than 8, which meet the criteria of normal distribution.

**TABLE 6:** Descriptive statistical analysis of pilot survey (*N* = 183).

	Item	Single mean	Overall mean	SD	Kurtosis	Skewness
Functional value (FV)	FV1	4.08	4.30	0.931	−1.073	1.210
	FV2	4.50		0.718	−1.903	5.443
	FV3	4.30		0.713	−1.064	2.158
Emotional value (EV)	EV1	4.05	4.04	0.768	−0.388	−0.427
	EV2	4.26		0.746	−0.789	0.266
	EV3	4.08		0.784	−0.492	−0.303
	EV4	3.77		0.848	−0.295	−0.474
Social value (SV)	SV1	4.14	4.15	0.742	−0.479	−0.286
	SV2	4.02		0.763	−0.262	−0.643
	SV3	4.34		0.707	−0.968	1.003
	SV4	4.11		0.726	−0.169	−1.079
Cognition value (CV)	CV1	4.26	4.19	0.693	−0.602	0.025
	CV2	4.23		0.722	−0.565	−0.273
	CV3	4.26		0.667	−0.458	−0.256
	CV4	3.99		0.832	−0.616	0.543
Self-accomplishment value (SAV)	SAV1	3.50	3.16	0.905	−0.124	−0.455
	SAV2	2.98		1.107	0.156	−0.427
	SAV3	2.99		1.066	0.121	−0.348
	SAV4	3.18		1.003	−0.006	−0.233

Data source: this study.

#### Reliability test

The reliability of the questionnaire was tested by the internal consistency coefficient and the overall correlation coefficient of the item (CITC). The Cronbach’s α coefficient and CITC value of each variable were obtained by using SPSS24.0 for the items of five latent variables: FV, EV, SV, CV, and SAV. The results showed that Cronbach’s α coefficient of the total scale was 0.914, and of the five subscales were 0.767, 0.784, 0.826, 0.871, and 0.927 respectively, which are all acceptable. The CITC value of each item in the study also reaches the standard of 0.5. However, the Cronbach’s α coefficient was significantly increased after the fourth item of CV, i.e., “*I can better reflect on and recognize my own culture through local knowledge tourism experience*,” was deleted. To ensure the internal consistency of the questionnaire, it is therefore decided to delete the item. The author argues that the reason why the deleted fourth item of the CV dimension failed to meet the standard is because the respondents in the interview were all Chinese, they may not be able to distinguish between their own culture and that of the others in the case of Guilin.

#### Exploratory factor analysis

To investigate the underlying dimensions of the items, an exploratory factor analysis (EFA) was performed with a component analysis and the varimax rotation method. The Kaiser-Meyer-Olkin test (KMO = 0.866) and Bartlett’s test of sphericity (χ^2^ = 5,860.12, df = 528; *p* < 0.001) indicated suitability to conduct the factor analysis. It was found that the remaining 18 items could be extracted into five dimensions, and the cumulative explained variance has reached 73.374%. The distribution of loading values for each factor after rotation was reported in Table 7 below.

**TABLE 7:** Exploratory factor analysis of the pilot survey questionnaire (*N* = 183).

Variable	Factor structure					
	**No.**	**SAV**	**SV**	**CV**	**EV**	**FV**
Functional value (FV)	FV1	0.299	0.094	0.092	0.053	0.735
	FV2	0.076	0.156	0.281	0.196	0.782
	FV3	−0.016	0.071	0.091	0.161	0.815
Emotional value (EV)	EV1	0.127	0.180	0.252	0.639	0.244
	EV2	−0.092	0.215	0.199	0.715	0.225
	EV3	0.137	0.163	0.283	0.776	0.119
	EV4	0.359	0.201	0.030	0.684	−0.034
Social value (SV)	SV1	0.101	0.629	0.272	0.229	0.219
	SV2	0.282	0.773	0.243	0.151	−0.036
	SV3	0.083	0.814	0.112	0.185	0.155
	SV4	0.307	0.703	0.124	0.198	0.088
Cognition value (CV)	CV1	0.103	0.214	0.807	0.284	0.127
	CV2	0.155	0.189	0.836	0.148	0.244
	CV3	0.160	0.234	0.824	0.247	0.131
Self-accomplishment value (SAV)	SAV1	0.811	0.247	0.100	0.155	0.086
	SAV2	0.874	0.143	0.062	0.075	0.101
	SAV3	0.896	0.120	0.117	0.124	0.103
	SAV4	0.889	0.152	0.156	0.061	0.082

Data source: this study. Highlights represent the items with loading value above 0.6, indicating good convergence validity, are combined to form a common factor.

After item CV4 has been deleted, the 18-item scale was finally formed. All factor loading is greater than 0.6 with most loading exceeding 0.7, which indicates that the samples have satisfactory convergence validity. The EFA results are basically consistent with the findings of the grounded theory coding, which preliminarily verifies that this newly developed local knowledge tourism perceived value scale has good reliability and validity.

### The formal survey to validate the scale

#### Investigation process and sample analysis

The sample size was finally established at 400 by considering important factors such as the size of Guilin City, the number of measured items, and the time period in which the survey is conducted. The survey was carried out in a mixed way. The face-to face survey was done at five major scenic areas of Guilin, including the East and West Alley, Longsheng Rice Terraces, Guangxi Ethnic Tourism Museum, etc. And the Internet survey was conducted with the online survey firm Wenjuanxing (a survey software) from June to July 2022. A total of 456 questionnaires were collected, including 256 from the site and 200 from the internet. After excluding questionnaires with short filling time and consistent answers for all questions, 412 valid questionnaires were finally obtained, with an effective rate of 90.35%. The sample size has fully reached the standard and can be used for subsequent confirmatory data analysis following the suggestions by Hair (2009). Only those who had a local knowledge tourism experience in Guilin over the last 12 months were invited for the survey. A total of 57.3% of the respondents were male and 93.7% are aged between 21 and 50, which indicates that young and middle-aged tourists show more interest in local knowledge tourism. Local knowledge tourism seems to be more popular among tourists with higher education background as 70.9% of the respondents had a college/university degree. This may be related to its knowledge-oriented attributes. Due to the ongoing COVID-19 pandemic, 57% of the respondents were from Guangxi and the rest mainly from the neighboring provinces.

#### Descriptive statistical analysis

The descriptive statistical analysis of the formal survey suggests that the overall mean values of FV, EV, SV, CV, and SAV were 3.98, 3.80, 3.79, 3.92, and 3.45, respectively. The SAV score was still relatively low, but higher than 3.16 in the pilot surveys. The data skewness coefficient of each measurement element is between −0.931 and −0.022 with an absolute value less than 2, and the kurtosis coefficient is between −0.943 and 1.481 with an absolute value less than 8, which indicates that samples are normally distributed. The factor analysis suggested that the explained variance of the first factor without rotation was 27.410%, smaller than 40%, and there was no large amount of explained variance concentrated in one factor, indicating that the influence of the deviation of the common method was small and acceptable.

#### Reliability and validity tests

Cronbach’s α coefficient and combined reliability CR value are used to test the reliability of each latent variable in the theoretical model to determine the consistency and stability of the data. As shown in Table 8, the Cronbach’s α coefficient of the total scale has reached 0.840, and ranges from 0.810 to 0.850 for each latent variable (>0.7). At the same time, the CR value of the combined reliability of each latent variable was between 0.803 and 0.850 (>0.7). According to the reliability test standard proposed by Fornell and Larcker (1981), the measurement of each latent variable has good reliability.

**TABLE 8:** Reliability test of the measurement model (*N* = 412).

	Item no.	Standardized factor load	AVE	CR	Cronbach’s α
Functional value (FV)	FV1	0.866	0.655	0.850	0.847
	FV2	0.783			
	FV3	0.776			
Emotional value (EV)	EV1	0.710	0.516	0.810	0.809
	EV2	0.703			
	EV3	0.755			
	EV4	0.704			
Social value (SV)	SV1	0.778	0.505	0.803	0.801
	SV2	0.666			
	SV3	0.748			
	SV4	0.643			
Cognition value (CV)	AV1	0.798	0.607	0.822	0.820
	AV2	0.723			
	AV3	0.813			
Self-accomplishment value (SAV)	MV1	0.664	0.548	0.828	0.825
	MV2	0.740			
	MV3	0.746			
	MV4	0.803			
The total scale					0.840

Data source: this study.

The validity test is conducted from two aspects: aggregate validity and discriminant validity. As can be seen from Table 9, the standardized factor loading of each measurement item is between 0.643 and 0.866 (>0.5)and AVE values of each measurement item ranges from 0.505 to 0.655 (>0.5), which indicates the good aggregate validity. Regarding the discriminant validity, the square root of the extracted average variance of each latent variable is higher than the correlation coefficient between each latent variable (see Table 10), suggesting that the discriminant validity is also satisfactory.

**TABLE 9:** Discriminant validity test (*N* = 412).

	FV	EV	SV	MV
FV	**0.809**			
EV	0.410***	**0.718**		
SV	0.345***	0.289***	**0.711**	
AV	0.285***	0.378***	0.285***	
MV	0.212***	0.299***	0.279***	**0.740**

Data source: this study. The bold value is the square root of the mean variance extraction of each variable. When it is greater than the correlation coefficient between any two variables, we can say that the discriminant validity between the latent variables is good.

***Represents *P* < 0.001, meaning that it is significant at the confidence level of 0.001.

**TABLE 10:** Fit results of the measurement model (*N* = 412).

Model fitting index	Testing data	Judgment threshold
χ^2^/DF	1.505	1 < χ^2^/DF < 3
RMR	0.033	RMR < 0.05
RMSEA	0.035	RMSEA < 0.08
GFI	0.951	0.9 < GFI < 1
AGFI	0.934	0.8 < AGFI < 1
NFI	0.936	NFI > 0.9
RFI	0.922	RFI > 0.9
PNFI	0.765	PNFI > 0.5
PGFI	0.696	PGFI > 0.5
IFI	0.978	IFI > 0.9
TLI	0.972	TLI > 0.9
CFI	0.977	CFI > 0.9

Data source: this study.

#### Test of the measurement model

This study constructed a five-factor model of tourist perceived value and used AMOS25.0 to conduct the confirmatory factor analysis. As illustrated in Table 10, χ^2^/df is 1.505, which is between 1 and 3, and significant at the level of *p* < 0.001. RMR is 0.033, less than 0.05; RMSEA is 0.035, less than 0.08. Other fitting indices such as GFI, AGFI, NFI, RFI, IFI, TLI, and CFI have all reached the corresponding standards, indicating that it is a satisfactory model.

Through the above steps of the confirmatory factor analysis, the items on the local knowledge tourism experience value measurement scale are finally confirmed as shown in Table 11.

**TABLE 11:** Items on the local knowledge tourism perceived value scale.

Construct	Item	Reference and source
Functional value (FV)	1. I can perceive the usage and function of local knowledge in daily life such as cuisine, living, traveling, and medical treatment on a tour	Na et al. (2019), interview and observation
	2. The local knowledge tourism experience makes me feel physically and mentally happy	
	3. This tour experience entails special financial, time, and energy costs	
Emotional value (EV)	4. I can feel the strong atmosphere created by the traditional culture, festivals, history, and legends of the tourist destination	Sheth et al. (1991), interview and observation
	5. I may associate the past era and the scene of people’s life at that time due to the local knowledge tourism experience	
	6. I am amazed at the miracle of local knowledge and traditional wisdom in the destination	
	7. I may have the feeling of identity of the identity of the place and attachment to the destination due to the local knowledge tourism experience	
Social value (SV)	8. Local knowledge tourism can help me make more friends	Ma and Bao (2012), interview and observation
	9. The local knowledge tourism experience makes me more willing to communicate and interact with the local people	
	10. Participating in local knowledge tours with friends and family can enhance mutual understanding and affection between us	
	11. The local knowledge tourism experience provides me with more information and knowledge about the destination	
Cognition value (CV)	12. I can better understand the unique history, tradition, culture, and art of the destination from the local knowledge tourism experience	Interview and observation
	13. I can better understand the unique ecological and natural environment of the destination through local knowledge tourism experience	
	14. I can better understand the unique social and humanistic environment of the destination through local knowledge tourism experience	
Self-accomplishment value (SAV)	15. Local knowledge tourism experience makes me a better self	Interview and observation
	16. Local knowledge tourism experience can enhance my social prestige	
	17. Local knowledge tourism experience enables me to realize my aspiration and ambition	
	18. Local knowledge tourism experience allows me to exert my potential	

Data source: this study.

## Discussion and conclusion

### Structure and characteristics of local knowledge tourism perceived values

Based on the theory of customer perceived value and the case study of Guilin, China, this study has first identified five dimensions of perceived value that include FV, EV, SV, CV, and SAV of local knowledge tourism using grounded theory. Later, rigorous procedures have been followed to develop the related scale that consists of 18 items as illustrated in Table 11 above. Related tests indicate that the newly developed scale is of satisfactory reliability and validity. This study attempts to investigate the perceived value of local knowledge by tourists by tourists to fill the gap, which is conducive to its conservation and development. The development of local knowledge as a tourist attraction can highlight the distinct characteristics and local spirit of the destination, helping to strengthen the competitiveness of the destination.

**FIGURE 1:**
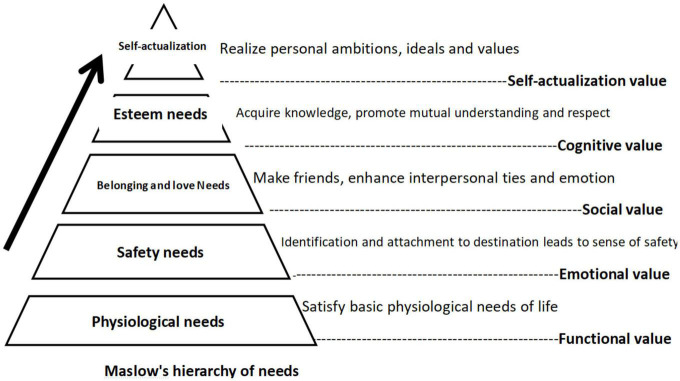
Hierarchical model of local knowledge tourism perceived values. Data source: this study.

Inspired by hierarchy of needs, Pearce and Caltabiano (1983) studied the progressive model of tourist demand and found that the higher the level of tourism motivation, the higher the demand will be. The local knowledge tourism perceived values identified by grounded theory in this study also showed corresponding progressive relationship between the tourist perceived values dimensions. As shown in Figure 1 below, the five identified local knowledge tourism perceived value dimensions form a corresponding internal relationship in line with the Maslow’s hierarchy of needs. The FV is reflected in the tourist perception that local knowledge can be utilized to meet the basic physiological needs such as food, accommodation and transportation. EV related to a variety of positive emotions that have been triggered during the tour. Local knowledge experience can stimulate tourists’ curiosity, association, and nostalgia, which may be transferred into emotions and feelings such as place identification and attachment to the destination and enhance tourists’ sense of belonging and safety. SV refers to social benefits such as making more new friends through the study of local knowledge in tourism activities. Other benefits may include the deepening of host–tourist interaction, closer ties between friends and improved family relationship, which is corresponding to the satisfaction of the belonging and love needs in the Maslow’s hierarchy. The main motivation of local knowledge tourism is to seek and acquire knowledge. As such, tourism activities will improve participants’ understanding of the destination with regards to its natural environment and traditional culture and promote mutual understanding and respect between hosts and guests. Finally, the value of self-actualization implies that some tourists manage to enhance their cultural literacy and realize their personal dreams and aspirations in the local knowledge tourism experience. This corresponds to the highest level of self-actualization in the Maslow’s hierarchy. As a tourism activity typically focusing on cultural experience, the perceived value of local knowledge tourism presents a systematization and multi-dimensional features. With the deepening of the tourism experience and host–tourist interaction, the perceived value of the tourist of the will evolve step by step from satisfaction of basic philological needs to the advanced level such as self-actualization to meet the emotional, social, and self-actualization needs of the tourist.

### Research contribution: theoretical implications

Perceived value, a vital concept in the field of quality consumer experience, has been extensively utilized in tourism research. This study introduces the novel concept of local knowledge tourism within the realm of heritage tourism, thereby expanding the research scope on non-heritage tourism and enhancing the understanding of perceived value in tourism contexts. Furthermore, the development of a new scale contributes to a deeper comprehension of the content, components, and unique characteristics of local knowledge tourism from the perspective of tourism experience and perceived value. This scale can serve as a valuable tool for future studies, such as investigating the impacts of perceived values related to local knowledge on tourist behaviors.

### Research contribution: managerial implications

From a management perspective, this study emphasizes the distinctive values of local knowledge in tourism development, fostering the advancement of cultural tourism products and enhancing the overall competitiveness of destinations. The identified perceived values of tourists provide insights into the diverse dimensions and levels of the local knowledge tourism experience, thereby facilitating market segmentation and the formulation of targeted marketing strategies for this niche cultural tourism market. For instance, the research categorizes local knowledge tourists into functional experience, emotional attachment, knowledge seeking and development, and self-actualization types. From the tourists’ standpoint, this study contributes to enhancing their travel experiences, increasing their engagement, and deepening their appreciation of the traditions and culture of the destination.

### Limitations and future research

Despite several contributions, this study still has some limitations. Firstly, local knowledge varies a lot in forms and types, and so does the tourist when it comes to their background, motivation, preference, and demand. To broaden this exploratory study, future research can be further conducted under different local knowledge tourism settings. The comparative study can be expanded to tourists of different age, occupation, education, and income level. Second, this research is carried out carried out primarily after the pandemic outbreak, the national border of China remains close to international tourists up to the end of the study. As a consequence, the interview participants and online travelogue used as secondary data were all Chinese. When China’s inbound markets is restored, the in-depth interviews can be conducted with international tourists of different cultural and social background. Furthermore, local knowledge tourism is diverse and influenced by various cultural factors, and the perceived values of tourists may vary across different destinations. Future research should include a wider range of cultural contexts to enhance the generalizability of our findings.

## Data availability statement

The original contributions presented in this study are included in the article/supplementary material, further inquiries can be directed to the corresponding author.

## Ethics statement

Ethical review and approval was not required for the study on human participants in accordance with the local legislation and institutional requirements. The ethics committee waived the requirement of written informed consent for participation.

## Author contributions

HZ: data curation, investigation, validation, and writing—review and editing. HZ and JZ: formal analysis, methodology, resources, software, visualization, and writing–original draft. JJ and JZ: funding acquisition, project administration, and supervision. All authors contributed to the conceptualization, article, and approved the submitted version.

## References

[B1] Andrades L. Dimanche F. (2018). “Co-creation of experience value: A tourist behaviour approach,” in *Creating experience value in tourism*, eds Prebensen N. K. Chen J. S. Uysal M. (London: CAB International Wallingford), 83–97.

[B2] Bao J. Xie H. J. (2019). Determinants of domestic tourism demand for Guilin. *J. China Tour. Res.* 15 1–14.

[B3] Behnam M. Anagnostopoulos C. Byers T. Papadimitriou D. A. (2023). The impact of perceived corporate social responsibility on value-in-use through customer engagement in non-profit sports clubs: The moderating role of co-production. *Eur. Sport Manage. Q.* 23 789–810. 10.1080/16184742.2021.1929375

[B4] Beritelli P. Bieger T. Laesser C. (2007). Destination governance: Using corporate governance theories as a foundation for effective destination management. *J. Travel Res.* 46 96–107.

[B5] Biao H. DengMing X. LianXin Z. Guo Q. (2020). Scale development and empirical validation of duty-free shopping tourist perceived value. *Tour. Tribune* 35 120–132.

[B6] Bunghez C. L. (2016). The importance of tourism to a destination’s economy. *J. Eastern Europe Res. Bus. Econ.* 2016 1–9.

[B7] Butler R. (2017). The tourist experience: Can destinations maintain authenticity? *Worldwide Hosp. Tour. Themes* 9 617–626.

[B8] Chaiboonsr C. Chokethaworn K. Wabg R. (2022). *Analyzing factors that effecting on tourism demand in Zhejiang tourist festival, based on 2019 Xitang Hanfu cultural festival using sem method.* Ph.D. Thesis. Chiang Mai: Chiang Mai University.

[B9] Chatzopoulou E. Gorton M. Kuznesof S. (2019). Understanding authentication processes and the role of conventions: A consideration of Greek ethnic restaurants. *Ann. Tour. Res.* 77 128–140. 10.1016/j.annals.2019.06.004

[B10] Chen C.-F. Chen F.-S. (2010). Experience quality, perceived value, satisfaction and behavioral intentions for heritage tourists. *Tour. Manage.* 31 29–35. 10.1016/j.tourman.2009.02.008

[B11] Chenini A. Touaiti M. (2018). Building destination loyalty using tourist satisfaction and destination image: A holistic conceptual framework. *J. Tour. Heritage Serv. Market.* 4 37–43.

[B12] Correia A. Kozak M. Ferradeira J. (2013). From tourist motivations to tourist satisfaction. *Int. J. Cult. Tour. Hosp. Res.* 7 411–424. 10.1108/IJCTHR-05-2012-0022

[B13] de Bruin A. Jelinčić D. A. (2016). Toward extending creative tourism: Participatory experience tourism. *Tour. Rev.* 71 57–66. 10.1108/TR-05-2015-0018

[B14] de Ruyter K. Wetzels M. Bloemer J. (1998). On the relationship between perceived service quality, service loyalty and switching costs. *Int. J. Serv. Ind. Manage.* 9 436–453. 10.1108/09564239810238848

[B15] Fornell C. Larcker D. F. (1981). Evaluating structural equation models with unobservable variables and measurement error. *J. Market. Res.* 18 39–50. 10.1177/002224378101800104

[B16] Gao J. Wu B. (2017). Revitalizing traditional villages through rural tourism: A case study of Yuanjia Village, Shaanxi Province, China. *Tour. Manage.* 63 223–233.

[B17] Gato M. Dias Á Pereira L. da Costa R. L. Gonçalves R. (2022). Marketing communication and creative tourism: An analysis of the local destination management organization. *J. Open Innov.* 8:40. 10.3390/joitmc8010040

[B18] Geertz C. (1974). “From the native’s point of view”: On the nature of anthropological understanding. *Bull. Am. Acad. Arts Sci.* 28 26–45.

[B19] Glover N. (2008). Co-produced histories: Mapping the uses and narratives of history in the tourist age. *Pub. Hist*. 30, 105–124. 10.1525/tph.2008.30.1.105

[B20] Grönroos C. (1997). Value-driven relational marketing: From products to resources and competencies. *J. Market. Manage.* 13 407–419. 10.1080/0267257X.1997.9964482

[B21] Groth J. (1995). Important factors in the sale and pricing of services. *Manage. Decis.* 33 29–34. 10.1108/00251749510090557

[B22] Hair J. F. (2009). *Multivariate data analysis.* Upper Saddle River, NJ: Pearson.

[B23] Halkier H. (2014). Innovation and destination governance in Denmark: Tourism, policy networks and spatial development. *Eur. Plann. Stud.* 22 1659–1670.

[B24] Hill R. Adem Ç Alangui W. V. Molnár Z. Aumeeruddy-Thomas Y. Bridgewater P. (2020). Working with Indigenous, local and scientific knowledge in assessments of nature and nature’s linkages with people. *Curr. Opin. Environ. Sustain.* 43 8–20. 10.1016/j.cosust.2019.12.006

[B25] Huang Y. Huang F. (2007). Tourists’ perceived value model and its measurement: An empirical study. *Tour. Tribune* 22 42–47.

[B26] Kim M. Thapa B. (2018). Perceived value and flow experience: Application in a nature-based tourism context. *J. Destin. Market. Manage.* 8 373–384. 10.1016/j.jdmm.2017.08.002

[B27] Klein K. J. Dansereau F. Hall R. J. (1994). Levels issues in theory development, data collection, and analysis. *Acad. Manage. Rev.* 19 195–229. 10.5465/amr.1994.9410210745

[B28] Lenzerini F. (2011). Intangible cultural heritage: The living culture of peoples. *Eur. J. Int. Law* 22 101–120. 10.1093/ejil/chr006

[B29] Lepore W. Hall B. L. Tandon R. (2021). The knowledge for change consortium: A decolonising approach to international collaboration in capacity-building in community-based participatory research. *Can. J. Dev. Stud.* 42 347–370.

[B30] Li J. Cheng S. Zhong L. (2009). Progress on the research of customer value theory in tourism. *Hum. Geogr.* 24 21–24.

[B31] Lim C. (2013). Analysis of time pressure and value perception: An exploratory study of consumer travel fair. *J. Travel Tour. Market.* 30 509–521. 10.1080/10548408.2013.803399

[B32] Loureiro S. M. C. Dias Sardinha I. M. Reijnders L. (2012). The effect of corporate social responsibility on consumer satisfaction and perceived value: The case of the automobile industry sector in Portugal. *J. Clean. Product.* 37, 172–178. 10.1016/j.jclepro.2012.07.003

[B33] Luo W. Tang P. Jiang L. Su M. M. (2020). Influencing mechanism of tourist social responsibility awareness on environmentally responsible behavior. *J. Clean. Prod.* 271:122565. 10.1016/j.jclepro.2020.122565

[B34] Lyu J. Krasonikolakis I. Chen C.-H. (2023). Unlocking the shopping myth: Can smartphone dependency relieve shopping anxiety? – A mixed-methods approach in UK Omnichannel retail. *Inf. Manage.* 60:103818. 10.1016/j.im.2023.103818

[B35] Ma L. Bao J. (2012). Traditional festival tourism experience from the perspective of perceived value—taking Xishuangbanna Dai water splashing festival as an example. *Geogr. Res.* 31 269–278.

[B36] Mills J. Bonner A. Francis K. (2006). The development of constructivist grounded theory. *Int. J. Qual. Methods* 5 25–35. 10.1177/160940690600500103

[B37] Moayerian N. McGehee N. G. Stephenson M. O. (2022). Community cultural development: Exploring the connections between collective art making, capacity building and sustainable community-based tourism. *Ann. Tour. Res.* 93:103355. 10.1016/j.annals.2022.103355

[B38] Na M. Xie Y. Gursoy D. (2019). A study on destination experiential value: Multi-dimensional analysis and scale development. *Tour. Tribune* 34 48–60. 10.19765/j.cnki.1002-5006.2019.12.009

[B39] Overstreet G. A. (1993). Creating a value in oversupplied markets: The case of Charlotesville, Vinia, hotels. *Cornell Hotel Restaur. Adm. Q.* 34 68–96. 10.1177/001088049303400513

[B40] Pearce P. L. Caltabiano M. L. (1983). Inferring travel motivation from travelers’, experiences. *J. Travel Res.* 22 16–20. 10.1177/004728758302200203

[B41] Pechlaner H. Beritelli P. Pichler S. Peters M. Scott N. R. (2015). *Contemporary destination governance: A case study approach.* Bingley: Emerald Group Publishing.

[B42] Petrick J. F. (2004). The roles of quality, value, and satisfaction in predicting cruise passengers’ behavioral intentions. *J. Travel Res.* 42 397–407. 10.1177/0047287504263037

[B43] Pi P. Liu X. Gao Y. (2016). Research on scale development of tourism destination customer experience value. *Value Eng.* 35 30–35. 10.14018/j.cnki.cn13-1085/n.2016.24.012

[B44] Pongponrat K. (2011). Participatory management process in local tourism development: A case study on fisherman village on Samui Island, Thailand. *Asia Pac. J. Tour. Res.* 16 57–73. 10.1080/10941665.2011.539391

[B45] Pongponrat K. Chantradoan N. J. (2012). Mechanism of social capital in community tourism participatory planning in Samui Island, Thailand. *Tourismos* 7:272.

[B46] Rahmanita M. (2018). “The aesthetics of nature tourism through the philosophical perspective of Immanuel Kant,” in *Proceedings of the 2nd international conference on tourism, gastronomy, and tourist destination (ICTGTD 2018)*, Jakarta.

[B47] Ramos H. (2015). Mapping the field of environmental justice: Redistribution, recognition and representation in ENGO press advocacy. *Can. J. Sociol.* 40 355–376.

[B48] Rasoolimanesh S. M. Dahalan N. Jaafar M. (2016). Tourists’ perceived value and satisfaction in a community-based homestay in the Lenggong valley world heritage site. *J. Hosp. Tour. Manage.* 26 72–81. 10.1016/j.jhtm.2016.01.005

[B49] Sánchez-Fernández R. Iniesta-Bonillo M. Ã (2007). The concept of perceived value: A systematic review of the research. *Market. Theory* 7 427–451. 10.1177/1470593107083165

[B50] Sheth J. N. Newman B. I. Gross B. L. (1991). Why we buy what we buy: A theory of consumption values. *J. Bus. Res.* 22 159–170. 10.1016/0148-2963(91)90050-8

[B51] Snoj B. Pisnik Korda A. Mumel D. (2004). The relationships among perceived quality, perceived risk and perceived product value. *J. Product Brand Manage.* 13 156–167. 10.1108/10610420410538050

[B52] Sofield T. H. B. Li F. M. S. Wong G. H. Y. Zhu J. J. (2017). The heritage of Chinese cities as seen through the gaze of Zhonghua Wenhua—‘Chinese common knowledge’: Guilin as an exemplar. *J. Herit. Tour.* 12 227–250. 10.1080/1743873X.2016.1243121

[B53] Sweeney J. C. Soutar G. N. (2001). Consumer perceived value: The development of a multiple item scale. *J. Retail.* 77 203–220. 10.1016/S0022-4359(01)00041-0

[B54] UNESCO (2017). *Local Knowledge, Global Goals.* Paris: UNESCO.

[B55] Woodruff R. B. (1997). Customer value: The next source for competitive advantage. *J. Acad. Market. Sci.* 25:139. 10.1007/BF02894350

[B56] Xie Y. Li M. (2009). Development of China’s outbound tourism and the characteristics of its tourist flow. *J. China Tour. Res.* 5 226–242.

[B57] Yin C.-Y. Poon P. Su J.-L. (2017). Yesterday once more? Autobiographical memory evocation effects on tourists’ post-travel purchase intentions toward destination products. *Tour. Manage.* 61 263–274. 10.1016/j.tourman.2017.02.014

[B58] Yuan C. Gan L. Zhuo H. (2022). Coupling mechanisms and development patterns of revitalizing intangible cultural heritage by integrating cultural tourism: The case of Hunan Province, China. *Sustainability* 14:12. 10.3390/su14126994

[B59] Zeithaml V. A. (1988). Consumer perceptions of price, quality, and value: A means-end model and synthesis of evidence. *J. Market.* 52 2–22. 10.1177/002224298805200302

[B60] Zhang H. Li L. Yang Y. Zhang J. (2018). Why do domestic tourists choose to consume local food? The differential and non-monotonic moderating effects of subjective knowledge. *J. Destin. Market. Manage.* 10 68–77. 10.1016/j.jdmm.2018.06.001

[B61] Zhang Y. Liu Z. (2012). “The research of using animation design to promote the tourism economy under the guilin folk cultural characteristics,” in *Proceedings of the 2012 National Conference on Information Technology and Computer Science*, Berlin.

[B62] Zhu J. Airey D. Siriphon A. (2021). Chinese outbound tourists as international consumer in Northern Thailand: A dynamic mobility perspective. *J. Consum. Cult.* 22:1469540521994318. 10.1177/1469540521994318

[B63] Zhu J. Rahmanita M. Asmaniati F. Oesman I. Rachman A. (2022). “Reflexivity on qualitative tourism research methodology,” in *Current Issues in Tourism, Gastronomy and Tourist Destination Research*, eds Oktadiana B. Rahmanita M. Suprina R. Pan J. (London: Taylor & Francis). 10.1186/s12992-015-0113-0

[B64] Zhu J. Siriphon A. (2019). Community-based tourism stakeholder conflicts and the co-creation approach: A case study of Longji terrace fields, PRC. *J. Mekong Soc.* 15 37–54.

[B65] Zhu J. J. Liu Z. Shen X. Shan L. Zhang X. (2023). Corporate social responsibility (CSR) in the service industry: A systematic review. *Front. Environ. Sci.* 11:1150681. 10.3389/fenvs.2023.1150681

